# Clinico-Biochemical Profiles and Outcomes in Acute Decompensated Heart Failure

**DOI:** 10.7759/cureus.93504

**Published:** 2025-09-29

**Authors:** Diksha Gupta, Sunita Gupta, Shanker Deep Sondhi

**Affiliations:** 1 General Medicine, Maharishi Markandeshwar Institute of Medical Sciences and Research, Ambala, IND

**Keywords:** acute decompensated heart failure, biochemical profile, clinical profile, lv ejection fraction (lvef), study outcome

## Abstract

Background

Acute decompensated heart failure (ADHF) is a major global health concern with rising prevalence in India, marked by earlier onset, high mortality, and frequent hospitalizations. This study aims to compare the risk factors, clinical and biochemical profiles, and in-hospital outcomes of ADHF patients with preserved versus reduced ejection fraction, addressing a key gap in Indian data.

Materials and methods

A cross-sectional observational study was conducted in the Department of Medicine at MM Institute of Medical Sciences and Research, Mullana, enrolling 100 consecutive patients aged ≥18 years with a confirmed diagnosis of heart failure according to the American College of Cardiology/American Heart Association/Heart Failure Society of America (2022 guidelines) (ACC/AHA/HFSA) 2022 definition. Patients with congenital heart disease, chronic cor pulmonale, or rheumatic heart disease were excluded. After informed consent, all participants underwent detailed history-taking, clinical examination, and investigations, and were followed until discharge or death. Data were analyzed using SPSS v26.0 with descriptive and inferential statistics, with p < 0.05 considered statistically significant.

Results

Overall, 52% of patients had heart failure with reduced ejection fraction (HFrEF) and 48% had heart failure with preserved ejection fraction (HFpEF), with a male predominance and a mean age of 62.3 years. Anemia and sepsis were common precipitating factors, while hypertension, obesity, and chronic obstructive pulmonary disease (COPD) were more associated with preserved ejection fraction (EF). In-hospital mortality was 10%. Elevated total leukocyte count (TLC), N-terminal pro-B-type natriuretic peptide (NT-proBNP), serum creatinine, and shock were significantly linked to poorer outcomes, with creatinine emerging as a strong predictor of mortality.

Conclusion

This study highlights the diverse clinico-biochemical profile of ADHF patients and the high prevalence of comorbidities. Significant differences were observed between preserved and reduced EF groups. Despite these variations, in-hospital mortality did not differ significantly, emphasizing the need for comprehensive risk assessment beyond ejection fraction to guide timely, individualized management in ADHF.

## Introduction

Acute decompensated heart failure (ADHF) refers to the sudden onset or worsening of heart-failure symptoms and clinical signs, often necessitating urgent medical intervention and frequently resulting in hospitalization. ADHF remains a growing public health concern due to its high incidence and significant impact on morbidity and mortality. In the United States, more than one million hospital admissions annually are attributed primarily to heart failure (HF), with an additional three million admissions in which HF is documented as a contributing diagnosis. It is a global health problem affecting 37.7 million people worldwide and 13-23 million people in the Indian subcontinent. The disease is associated with increased mortality over both 1- and 5-year periods. Indians suffer from HF approximately 10 years earlier than Western populations, with a substantial economic burden. The prevalence of HF continues to rise in India, primarily driven by an increase in cardiovascular risk factors. The disease burden is projected to increase by 25% by 2030 [1,2].

Over the decades, the classification of HF has evolved with advances in clinical understanding, diagnostic modalities, and therapeutic approaches. The earliest attempt was the New York Heart Association (NYHA) classification introduced in 1928, which categorized patients based on the degree of functional limitation. Later, in the 1970s and 1980s, hemodynamic classifications emerged, based on invasive monitoring of cardiac output and filling pressures, introducing terms such as high-output vs low-output and forward vs backward failure [3]. A major shift occurred with the widespread use of echocardiography, leading to an ejection-fraction (EF)-based classification. In the 1980s and 1990s, left ventricular ejection fraction (LVEF) became a widely accepted quantitative parameter to differentiate types of HF. This led to the definition of heart failure with reduced ejection fraction (HFrEF) as LVEF <40%. Recognition of heart failure with preserved ejection fraction (HFpEF) came in the early 2000s. Earlier, HFpEF was defined as EF ≥50%, while EF 41-49% was termed “heart failure with mid-range EF (HFmrEF).” However, newer evidence and guideline updates emphasize that the EF spectrum is continuous, and patients with EF >40% share similar clinical characteristics, pathophysiology, and outcomes compared with those with EF ≥50% [4].

HFpEF and HFrEF differ not only in pathophysiology and underlying comorbidities but also in clinical presentation, biochemical profiles, response to therapy, and outcomes. The differentiation between HFrEF and HFpEF has important diagnostic, therapeutic, and prognostic implications. The clinico-biochemical parameters of Indian populations may differ from those of Western populations due to variations in risk factors, socioeconomic status, healthcare access, and disease awareness [5]. A detailed understanding of the clinico-biochemical profile in ADHF can aid in early diagnosis, appropriate risk stratification, and timely therapeutic interventions. There are very few studies directly comparing patients with HFrEF versus those HFpEF while highlighting risk factors, precipitating factors, outcomes, and management. In light of this gap, the present study aims to evaluate and compare the underlying risk factors and the clinical and biochemical profiles of ADHF with preserved ejection fraction versus reduced ejection fraction, and to assess in-hospital outcomes.

## Materials and methods

The study aimed to evaluate the clinical and biochemical profile, as well as the in-hospital outcomes, of patients with ADHF. The study was conducted from March 2024 to March 2025 in the Department of Medicine, Maharishi Markandeshwar Institute of Medical Sciences and Research, Ambala. It included 100 consecutive eligible patients who met the inclusion criteria.

Inclusion criteria

Patients aged 18 years and above with a confirmed diagnosis of HF according to the ACC/AHA/HFSA 2022 definition [6].

Exclusion criteria

Patients with (a) congenital heart disease, (b) chronic cor pulmonale, (c) rheumatic heart disease, or (d) end-stage kidney disease.

The Institutional Ethics Committee of Maharishi Markandeshwar Institute of Medical Sciences & Research, Mullana, approved the study design (IEC-2872).

The selected patients were briefed about the nature of the study, and written informed consent was obtained from them in the regional language. Detailed history and clinical examination were performed for all patients. Laboratory parameters, complete blood count (CBC), liver function test (LFT), renal function test (RFT), random blood sugar (RBS), pro-brain natriuretic peptide (pro-BNP), ECG, chest radiograph (chest X-ray), and 2D echocardiography were obtained for all selected patients. Echocardiography for ejection-fraction measurement was performed using a Philips EPIC 7C machine. Measurements were obtained by the anterior-lateral (AL) method. Three echocardiographic measurements of ejection fraction (EF) were taken by the same observer, and the mean of those three readings was reported.

Guideline-directed medical therapy included angiotensin-converting enzyme (ACE) inhibitors or angiotensin receptor blockers (ARB) or angiotensin receptor-neprilysin inhibitors (ARNI), beta-blockers, decongestants (loop diuretics such as furosemide or torsemide, or mineralocorticoid receptor antagonists (MRA)), inotropic support (dobutamine, dopamine), and antiarrhythmics, with the drug of choice and dosing individualized as per patient profile [7,8]. Patients were monitored until discharge or mortality.

Operational definitions

Sepsis was defined as a life-threatening organ dysfunction caused by a dysregulated host response to infection, with an acute increase in the Sequential Organ Failure Assessment (SOFA) score of ≥2 points.

Anemia was defined based on WHO criteria, with a hemoglobin concentration of <13 g/dL in men and <12 g/dL in non-pregnant women taken as the diagnostic cutoff (WHO, 2011).

COPD was defined and classified according to the 2025 Global Initiative for Chronic Obstructive Lung Disease (GOLD) guidelines.

Obesity was defined according to the WHO Asian-specific BMI cutoffs [9-12].

Statistical analysis

All 100 patients were divided into two groups on the basis of EF: those with EF >40% were included in the preserved ejection-fraction group, and those with EF <40% in the reduced ejection-fraction group. Patients with mildly reduced ejection fraction (41-49%) were combined with the preserved ejection-fraction group, as they had similar characteristics to those of the preserved group. The analysis included demographic profile and clinical and laboratory parameters. Descriptive analysis of quantitative parameters was expressed as mean and SD. Categorical data were expressed as absolute number and percentage. The independent Student’s t-test was used to compare means between independent groups. A p-value <0.05 was considered statistically significant. All analyses were performed using SPSS software, version 26.0. A 95% CI was considered for all estimates.

## Results

We divided all subjects into two groups: reduced EF (52%) and preserved EF (48%). Males predominated (63 males, 37 females). Table 1 shows no statistically significant age or gender differences between the HFrEF and HFpEF groups. More elderly patients were found in the preserved EF group (p = 0.326), and patients with HFpEF had significantly higher body weight and BMI (p < 0.001). Among clinical features, a significant difference was noted in peripheral edema, which was more frequent in HFpEF than in HFrEF (p = 0.004).

**Table 1:** Comparison of the demographic and clinical profiles of HFpEF and HFrEF. HFrEF: Heart Failure with Reduced Ejection Fraction; HFpEF: Heart Failure with Preserved Ejection Fraction; JVP: Jugular Venous Pressure. *Significant at p < 0.05.

Variables	Reduced EF group (n=52)	Preserved EF group (n=48)	Chi-square value / T-statistic	p-value
	Number (%)/ Mean (±SD)	Number (%)/ Mean (±SD)		
Males	32 (61.54%)	31 (64.58%)	0.012	0.753
Females	20 (38.46%)	17 (35.42%)		
Weight (kg)	65.5 (±9.21)	75.32 (±11.36)	-4.18	0.000*
BMI (kg/m²)	25.83 (±3.66)	30.12 (±4.64)	-4.6	0.000*
Mean age (years)	60.73 (±14.04)	63.85 (±12.01)	-0.45	0.326
Dyspnea	50 (96.15%)	41 (85.42%)	2.325	0.061
Fever	5 (9.62%)	8 (16.67%)	0.562	0.294
Chest pain	3 (5.77%)	7 (14.58%)	1.287	0.142
Pedal edema	4 (7.69%)	6 (12.50%)	0.218	0.336
Cough	9 (17.31%)	3 (6.25%)	1.938	0.089
Generalized swelling	1 (1.92%)	1 (2.08%)	0	0.95
Palpitations	1 (1.92%)	0 (0%)	0	0.334
Vomiting	0 (0%)	1 (2.08%)	0.002	0.295
Peripheral edema	25 (48.08%)	38 (79.17%)	9.059	0.004*
Raised JVP	33 (63.46%)	32 (66.67%)	0.016	0.736
Crepitations / Wheeze	47 (90.38%)	45 (93.75%)	0.063	0.535

Table 2 shows that anemia was the most common risk factor, found in 70 patients, and sepsis was present in 28 patients. The most common comorbidities were hypertension (53 patients) and obesity (47 patients). Diabetes mellitus was present in 41 patients, with 29 having both diabetes and hypertension. Other important comorbidities included old CAD (33), COPD (25), and ACS (7). Hypertension and COPD were significantly more common in the HFpEF group compared with the HFrEF group (p = 0.002 and p = 0.021, respectively). Old CAD was more frequent in HFrEF, though the difference did not reach statistical significance (p = 0.102).

**Table 2:** Comparison of comorbidities among patients with reduced and preserved EF. HFrEF: Heart Failure with Reduced Ejection Fraction; HFpEF: Heart Failure with Preserved Ejection Fraction; HTN: Hypertension; CAD: Coronary Artery Disease; ACS: Acute Coronary Syndrome; COPD: Chronic Obstructive Pulmonary Disease; TB: Tuberculosis. *Significant at p < 0.05.

Variables	Reduced EF (n = 52)	Preserved EF (n = 48)	Chi-square value	p-value
	Number (%)	Number (%)		
Hypertension (HTN)	20 (38.46%)	33 (68.75%)	8.017	0.002*
Diabetes	22 (42.31%)	19 (39.58%)	0.005	0.578
Diabetes + HTN	15 (28.85%)	14 (29.17%)	0	0.974
Sepsis	15 (28.85%)	13 (27.08%)	0	0.845
Anemia	33 (63.46%)	37 (77.08%)	1.604	0.289
Old CAD	21 (40.38%)	12 (25.00%)	2.021	0.102
ACS	4 (7.69%)	3 (6.25%)	0	0.777
COPD	8 (15.38%)	17 (35.42%)	4.327	0.021*
Old TB	1 (1.92%)	2 (4.17%)	0.005	0.511

Table 3 shows that patients with HFpEF had significantly higher serum creatinine (1.91 ± 0.95 vs. 1.12 ± 0.88 mg/dL, p = 0.002), higher pro-BNP levels (7,227.7 ± 6,210.2 vs. 5,949.3 ± 4,679.8 pg/mL, p < 0.001), and lower hemoglobin (9.2 ± 2.4 vs. 11.4 ± 2.4 g/dL, p = 0.002) compared with HFrEF. Sepsis, tachycardia, and shock were also more frequently seen in HFrEF, though the differences did not reach statistical significance.

**Table 3:** Comparison of hemodynamic, hematological, and biochemical profiles among patients with reduced and preserved EF. HFrEF: Heart Failure with Reduced Ejection Fraction; HFpEF: Heart Failure with Preserved Ejection Fraction; bpm: Beats per minute; SBP: Systolic Blood Pressure; DBP: Diastolic Blood Pressure; Hb: Hemoglobin; TLC: Total Leukocyte Count; PLT: Platelet Count; BNP: B-type Natriuretic Peptide. *Significant at p < 0.05.

Variables	Reduced EF group (n = 52)	Preserved EF group (n = 48)	Chi-square value / T-statistic	p-value
	Number (%)/ Mean (±SD)	Number (%)/ Mean (±SD)		
Pulse (bpm)	99.23 (±23.21)	91.52 (±16.26)	2.535	0.059
SBP (mmHg)	117.69 (±27.69)	136.88 (±28.15)	-1.364	0.001*
DBP (mmHg)	70.19 (±17.54)	76.08 (±17.14)	-2.449	0.093
Hb (g/dL)	11.41 (±2.38)	9.23 (±2.42)	4.459	0.002*
TLC (×1000/µL)	10.70 (±5.74)	11.05 (±3.82)	-0.589	0.722
PLT (×1000/µL)	242.12 (±87.11)	216.04 (±94.53)	1.862	0.154
Creatinine (mg/dL)	1.12 (±0.88)	1.91 (±0.95)	-4.063	0.002*
Sodium (mEq/L)	139.02 (±4.25)	138.10 (±6.95)	1.066	0.422
Pro-BNP (pg/mL)	5949.29 (±4679.75)	7227.68 (±6210.18)	0.62	<0.001*
Tachyarrhythmia	7 (13.54%)	3 (6.25%)	0.752	0.356

Table 4 shows that overall in-hospital mortality in the cohort was 10%, with 7.7% mortality in HFrEF and 12.5% in HFpEF; the difference between the two groups was not statistically significant (p = 0.423).

**Table 4:** Comparison of outcomes among patients with reduced and preserved EF. HFrEF: Heart Failure with Reduced Ejection Fraction; HFpEF: Heart Failure with Preserved Ejection Fraction.

Variables	Reduced EF group (n = 52)	Preserved EF group (n = 48)	Chi-square value	p-value
	Number (%)	Number (%)		
Collapsed	4 (7.69%)	6 (12.50%)	0.218	0.423
Survived	48 (92.31%)	42 (87.50%)		
Total	52 (100%)	48 (100%)		

Table 5 shows that patients who died (mortality group) had significantly lower systolic blood pressure (95 ± 30.6 vs. 125.1 ± 26.4 mmHg, p = 0.001) and diastolic blood pressure (59 ± 21.8 vs. 74.6 ± 16.4 mmHg, p = 0.007) compared with survivors. Total leukocyte count was also markedly higher in the mortality group (16.7 ± 5.7 vs. 10.2 ± 4.4 × 10³/cumm, p < 0.001). Serum creatinine levels were significantly higher among non-survivors (2.25 ± 0.9 vs. 1.6 ± 0.9 mg/dL, p = 0.034), indicating worse renal dysfunction. Similarly, pro-BNP levels were substantially elevated in the mortality group (11,201 ± 7,166 vs. 5,514 ± 4,947 pg/mL, p < 0.001).

**Table 5:** Correlation of outcomes with clinical and laboratory variables. bpm: Beats per minute; SBP: Systolic Blood Pressure; DBP: Diastolic Blood Pressure; TLC: Total Leukocyte Count; BNP: B-type Natriuretic Peptide. *Significant at p < 0.05.

Variable	Collapsed (n = 10)	Survived (n = 90)	Chi-square value / T-statistic	p-value
	Number (%)/ Mean (±SD)	Number (%)/ Mean (±SD)		
Pulse (bpm)	99 (±27.82)	95.14 (±19.62)	-0.059	0.574
SBP (mmHg)	95 (±30.64)	125.11 (±26.36)	-3.981	0.001*
DBP (mmHg)	59 (±21.83)	74.58 (±16.38)	-3.881	0.007*
TLC (×1000/µL)	16.70 (±5.73)	10.22 (±4.35)	3.901	0.000*
Anemia	7 (70.0%)	63 (70.0%)	0	1
Creatinine (mg/dL)	2.25 (±0.92)	1.60 (±0.91)	1.4	0.034*
Pro-BNP (pg/mL)	11201.30 (±7166.31)	5514.21 (±4947.09)	1.382	0.000*

**Figure 1:**
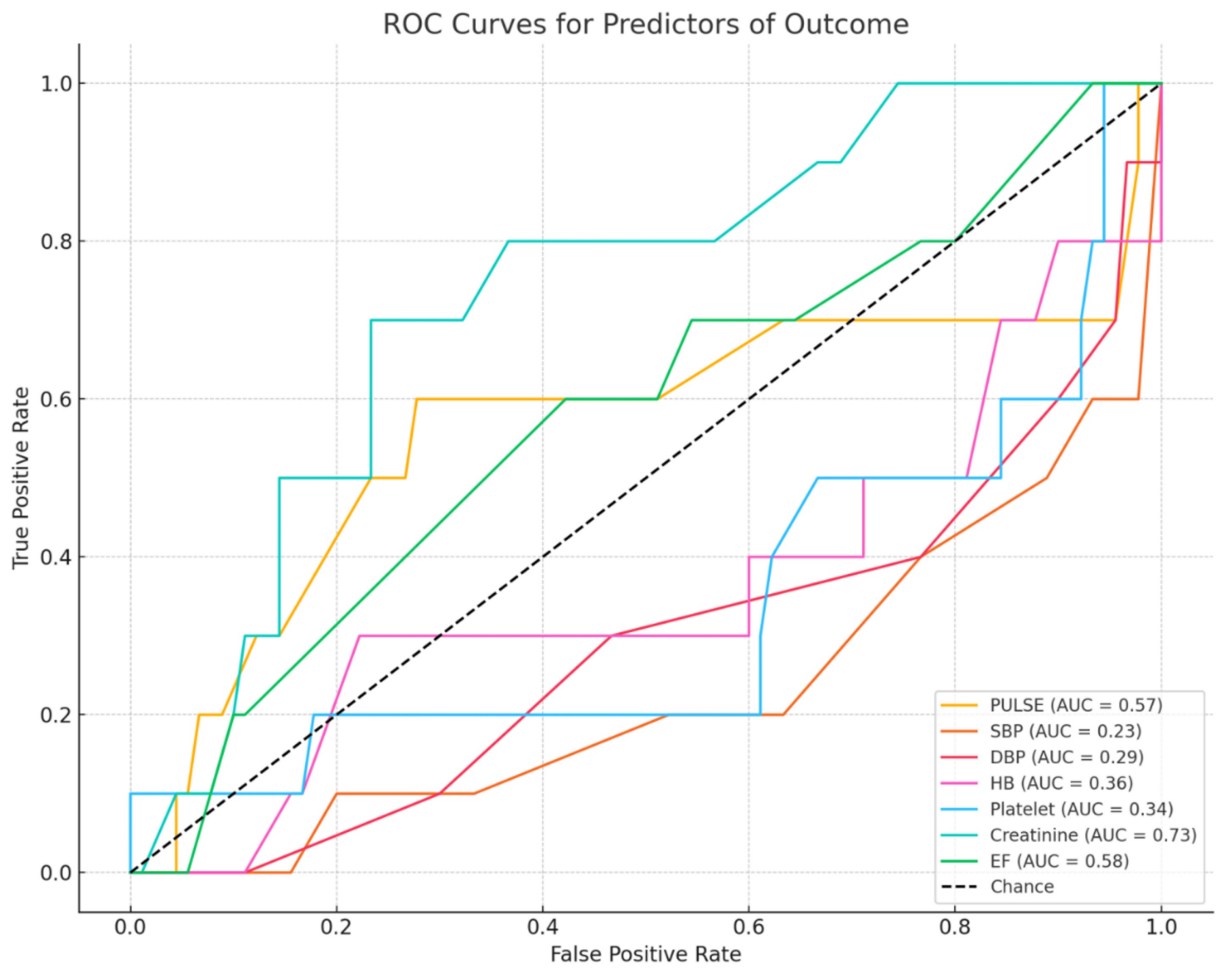
ROC curve for predictors of outcome. ROC: Receiver Operating Characteristic; AUC: Area Under the Curve; SBP: Systolic Blood Pressure; DBP: Diastolic Blood Pressure; Hb: Hemoglobin; EF: Ejection Fraction.

ROC analysis shows serum creatinine demonstrated the highest predictive ability for adverse outcomes, with an AUC of 0.73, indicating good discriminatory power (Figure 1).

## Discussion

In our study, the majority of patients were aged >60 years, reflecting the typical demographic profile of HF. The mean age was 62.3 years. However, nowadays, HF is increasingly observed in the younger population. In our study, 7% of patients were aged ≤40 years. Males constituted the majority across all age groups. This aligns with prior population-based studies demonstrating that HF primarily affects older adults, with men more frequently affected due to the higher prevalence of ischemic heart disease in males [13].

In our study, 52% of patients were in the HFrEF group and 48% were in the HFpEF group. The distribution between HFrEF and HFpEF was nearly equal, reflecting the growing recognition that HFpEF is increasingly prevalent among hospitalized HF patients.

Table 1 shows that the mean age was slightly higher in the HFpEF group compared to the HFrEF group (63.85 vs. 60.73 years), although this difference was not statistically significant. No significant gender differences were observed between the two groups. Consistent with these observations, Shah SJ et al. reported that HFpEF accounts for nearly half of all HF cases, with its prevalence rising in parallel with aging and comorbidities [14].

Table 1 shows that dyspnea (91%) was the predominant symptom, followed by cough, fever, and chest pain in our study. Edema was statistically significant in patients with preserved EF (p = 0.004) compared to the reduced EF group. Literature also supports the observation that systemic congestion, including edema and orthopnea, is more pronounced in HFpEF, as suggested by Mentz RJ et al. in his study [15].

Comorbidities play a crucial role in shaping the clinical presentation and outcomes of HF. Hypertension, diabetes, and obesity were common in our study, mirroring global trends. Obesity is a known risk factor for HFpEF and contributes to disease progression via mechanisms such as inflammation and diastolic dysfunction. In our study, patients with preserved EF had a statistically significantly higher BMI (p < 0.001). Pandey A et al. reported obesity as a common comorbidity in HFpEF patients [16].

Similarly, anemia emerged as a frequent precipitating factor in 70 (70%) patients, a finding that may be more pronounced in the Indian population due to dietary patterns and socioeconomic influences. Previous studies have consistently demonstrated the adverse prognostic implications of anemia in HF. Beale AL et al., Pan J et al., and Majmundar M et al. studied the prevalence of anemia in HF patients and its association with worse outcomes [17-19].

Infective etiologies, particularly sepsis in 28 (28%) patients, were also observed as important precipitants of decompensation. This aligns with reports from Krumholz HM et al. [20], who identified infection as a common trigger for hospitalizations in HF. These findings reinforce the view that HF, especially HFpEF, should be considered a systemic disorder of multimorbidity rather than a purely cardiac condition, consistent with findings by Yancy CW et al., Sharma A et al., and Paulus WJ and Tschöpe C [21-23]. These comorbidities exacerbate HF outcomes and reflect its complex clinical landscape, illustrating the multimorbidity nature of HF patients.

Table 3 shows that sepsis, tachycardia, and shock were more frequent in the reduced EF group, whereas raised creatinine, lower hemoglobin, and higher Pro-BNP levels were observed in the preserved EF group. Prior studies have shown that systemic markers such as blood pressure, creatinine, and BNP reflect the severity of decompensation and predict adverse outcomes, consistent with studies by Damman K et al. and McKie PM et al., who noted that higher BNP in HFpEF reflects elevated filling pressures [24,25]. Zhu JW et al. [26] highlighted that HFrEF patients commonly require inotropes and vasopressors due to hypotension and septic shock, while Damman K et al. [24] confirmed the high prevalence of renal dysfunction in acute decompensated HF.

Table 4 shows that hospital mortality in all patients with acute decompensated HF was 10%. Patients with preserved ejection fraction had a trend toward longer hospital stay compared to those with reduced EF (8.23 vs. 6.67 days), though this difference did not reach statistical significance (p = 0.059). About 10% of patients required inotropes or ventilatory support during admission. One patient required defibrillation for ventricular tachycardia. None of the patients required an implantable cardioverter-defibrillator (ICD) or cardiac resynchronization therapy (CRT). No significant difference in outcomes during the hospital stay was found between the two groups (p = 0.423). Thus, EF may not be the sole predictor of short-term outcomes, as other contributing factors such as sepsis, anemia, AKI, and shock also significantly influence HF outcomes.

Table 5 shows that mortality correlated with shock, sepsis, high Pro-BNP, and increased creatinine, markers of poor perfusion and end-organ damage.

Figure 1 depicts the ROC analysis demonstrating that serum creatinine (AUC = 0.73) is a reliable predictor of poor outcomes, consistent with Felker GM and Mentz RJ [27], who identified creatinine as a strong predictor of in-hospital mortality. The ROC curve further shows a negative correlation with systolic and diastolic blood pressure, indicating that hypotension and shock are strongly associated with adverse outcomes.

Our study has certain limitations that should be acknowledged. First, it was conducted in a single tertiary care center, which may restrict the generalizability of the findings to other populations and healthcare settings. Second, the relatively small sample size of 100 patients limits the statistical power to detect subtle but clinically meaningful differences between groups. Finally, the analysis was confined to in-hospital outcomes, and the lack of long-term follow-up precludes assessment of post-discharge morbidity and mortality.

## Conclusions

The present study highlights that, despite differences in the clinico-biochemical profiles of patients presenting with ADHF, in-hospital mortality and overall survival did not differ significantly between the two groups. This suggests that EF alone may not be a reliable predictor of short-term outcomes. These findings underscore the importance of a comprehensive assessment extending beyond EF for effective risk stratification and timely intervention in ADHF. Tailoring management strategies based on integrated clinical and biochemical markers may help improve outcomes and reduce morbidity associated with acute heart failure exacerbations.
